# Preventing pre-eclampsia – are dietary factors the key?

**DOI:** 10.1186/s12916-014-0176-4

**Published:** 2014-09-22

**Authors:** Jodie M Dodd, Cecelia O’Brien, Rosalie M Grivell

**Affiliations:** School of Paediatrics and Reproductive Health, and The Robinson Research Institute, The University of Adelaide, Adelaide, Australia; Department of Perinatal Medicine, Women’s and Babies Division, The Women’s and Children’s Hospital, North Adelaide, Australia

**Keywords:** Pre-eclampsia, Hypertension, Systematic review, Randomised trials, Dietary factors

## Abstract

Pre-eclampsia is a common pregnancy related condition, which contributes significantly both to maternal and perinatal morbidity and mortality. The precise pathophysiology of pre-eclampsia is uncertain, and the development of effective preventive strategies remains elusive.

Schoenaker and colleagues conducted a systematic review and meta-analysis of observational studies reporting dietary intake and dietary patterns. The findings indicated that women with a low dietary calcium intake were more likely to be diagnosed with gestational hypertension, while there was a suggestion (although not statistically significant) of a beneficial effect of a diet rich in fruits and vegetables on risk of pre-eclampsia.

This is in contrast to the findings of systematic reviews and meta-analyses of randomised trials in pregnancy evaluating calcium supplementation and anti-oxidant vitamin C and E supplementation.

The validity of any systematic review is reliant on both the underlying methodology and the quality of each of the included studies; the review by Schoenaker and colleagues is limited by the observational nature of the included studies.

Please see related article: http://www.biomedcentral.com/1741-7015/12/157/abstract.

## Background

Pre-eclampsia is a condition specific to the second half of pregnancy, affecting multiple organ systems, [1] and affecting approximately 2% to 8% of pregnant women [2]. While the condition is more commonly encountered among women in their first ongoing pregnancy, there are a number of additional recognised risk factors, including a past history of pre-eclampsia, underlying essential hypertension, the coexistence of autoimmune conditions and multi-fetal pregnancies [3]. While the incidence of disease risk factors has steadily increased, particularly an increase in maternal age at the time of first pregnancy and an increase in maternal obesity [3], the actual incidence of pre-eclampsia has decreased, reflecting, in part, a trend to an earlier gestational age at birth [4].

### Pre-eclampsia associated maternal morbidity and mortality

World-wide, hypertension during pregnancy contributes significantly to both maternal and perinatal mortality and morbidity [4-6], accounting for 10% to 15% of all direct maternal deaths [7]. The vast majority of maternal deaths attributable to pre-eclampsia occur in low to middle income countries [8], although the proportional contribution to direct maternal deaths in high income countries remains similar [9,10]. Pre-eclampsia is a contributing factor in up to one third of all cases of serious maternal morbidity, with 5% of women with severe disease requiring admission to intensive care [11,12], and in the longer-term, increases a woman’s risk of both chronic hypertension and cardiovascular disease [13,14].

### Pre-eclampsia associated perinatal morbidity and mortality

Independent of the effects of both prematurity and intra-uterine growth restriction, pre-eclampsia is a contributing factor in up to 2.7% of all perinatal deaths [15]. Furthermore, pre-eclampsia is documented as an antecedent factor in up to 12% of infants born with intra-uterine growth restriction [16] and 19% born preterm [17]. Both prematurity and small for gestational age are associated with an increased risk of neonatal complications, including respiratory distress syndrome, feeding difficulties, hypoglycaemia, seizures, intracranial haemorrhage and prolonged hospitalisation [18]. In the longer-term, preterm and small for gestational age infants remain at increased risk of neurodevelopmental delay in childhood [19], and subsequent obesity, insulin resistance and cardiovascular disease in later life [20].

### Strategies to prevent pre-eclampsia: are dietary strategies the key?

The recent systematic review and meta-analysis by Schoenaker and colleagues [21] reports an association between gestational hypertensive conditions, including pre-eclampsia, and dietary factors, based on observational studies. The review inclusion criteria were studies where energy intake, nutrient intake, food groups, or overall dietary patterns, either alone or in combination with dietary supplements, were reported, but excluded purely supplement-based studies. The findings of the review indicated that women with a low dietary calcium intake were more likely to be diagnosed with gestational hypertension, and while there were no significant associations between dietary content of vitamins C and E, vitamin D and n-3 polyunsaturated fatty acids with pre-eclampsia, there was a suggestion (although not statistically significant) of a beneficial effect of a diet rich in fruits and vegetables on risk of pre-eclampsia.

### How do these findings compare with the randomised trial literature evaluating strategies for the prevention of pre-eclampsia?

In general terms, the development of effective strategies for the prevention of pre-eclampsia has proven exceedingly difficult [5], related in part to the uncertainty of the precise ‘cause’ of the condition [1], and its likely multifactorial and complex nature. Many interventions have been proposed and evaluated in the prevention of pre-eclampsia, although low-dose aspirin [22] and calcium supplementation [23] remain the only strategies associated with a definitive reduction in risk. The use of antiplatelet agents has been associated with a 17% reduction in the risk of pre-eclampsia, and a modest 8% and 10% reduction in both preterm birth and small for gestational age infants, respectively [22]. While calcium supplementation has been associated with a relative risk reduction of 55% in pre-eclampsia, this appears to be largely confined to women with low dietary calcium intake [23]. Furthermore, the effects of calcium supplementation on improving measures of infant health are less clear [23].

The review by Schoenaker and colleagues [21] demonstrated no significant associations between dietary content of vitamins C and E, vitamin D and n-3 polyunsaturated fatty acids with pre-eclampsia. However, there was a suggested potential beneficial effect from a diet rich in fruits and vegetables, foods well recognised to be a rich source of vitamin C in particular. There has been considerable research interest in supplementation with anti-oxidant vitamins C and E as a potential strategy for the prevention of pre-eclampsia. Unfortunately, however, well conducted randomised trials and subsequent meta-analyses have demonstrated no beneficial effect of anti-oxidant vitamin supplementation in reducing a woman’s risk of developing pre-eclampsia [24].

### Why is there a discrepancy in findings?

Well-conducted systematic reviews and meta-analyses are highly regarded as a reliable source of information in the evidence hierarchy [25]. While the validity of any systematic review is heavily dependent on the underlying methodology, the certainty of the conclusions is limited by the quality of each of the individual studies identified and included. The review by Schoenaker and colleagues [21] is, therefore, limited by the observational nature of the included studies. Observational studies are able to identify associations, but randomised trials are required to evaluate cause and effect. Furthermore, the majority of the included observational studies utilised self-reported dietary questionnaires, which are subject to recall bias, and most were not validated. Most of the included studies did not adjust for important confounding factors, including maternal age, parity and pre-existing hypertension, all of which have been recognised to increase a woman’s risk of gestational hypertension and pre-eclampsia. There was substantial heterogeneity across the studies, particularly in relation to women’s unadjusted calcium and total daily energy intake. All of these factors limit the confidence with which any conclusions can be made.

As Schoenaker and colleagues [21] highlight, there is a need for well-powered prospective intervention studies to evaluate the role of healthy dietary patterns in pregnancy and their impact on maternal and infant health outcomes, including gestational hypertension and pre-eclampsia. Such a study would require a large sample size of the order of several thousand pregnant women, and dietary intervention studies are by necessity complex, difficult to conduct well and difficult to evaluate. This reflects the fact that ‘diet’ itself is complex, and it is not only individual dietary components that may impact disease pathophysiology, but also their combination – we tend to eat ‘food’ rather than individual macro- and micronutrients in isolation.

## Conclusions

It has been stated that: ‘Evidence does not speak for itself – it requires interpretation in light of its original context (and) limitations…’ [26]. The findings of Schoenaker’s systematic review [21] suggest a non-statistically significant benefit in pregnant women adopting a healthy dietary pattern. While acknowledging the limitations and observational nature of the included studies, this would seem to be prudent advice.
